# Combined effects of a pyrethroid insecticide and azole fungicide on lepidopteran growth analysed with DEB-TKTD modelling

**DOI:** 10.1007/s10646-025-02989-3

**Published:** 2025-12-09

**Authors:** Claire Badder, Sylvain Bart, Alex Robinson, Stephen Short, David J. Spurgeon

**Affiliations:** 1 Centre for Ecology and Hydrology, MacClean Building, Benson Lane, Crowmarsh Gifford, Wallingford, Oxon, OX10 8BB UK; 2 MO-ECO2 (Modelling and data analyses for ecology and ecotoxicology), Paris, France

**Keywords:** Insecticide, Fungicide, Mixture toxicity, Dynamic energy budget, Synergism

## Abstract

The co-application of insecticides and fungicides is common in crop protection. Several studies in bees have identified synergism between pyrethroid insecticides and azole fungicides for mortality and sublethal endpoints. However, there are no studies that detail this synergism in Lepidopteran species. Here, we conduct a mixture exposure with cypermethrin and the fungicide prochloraz to assess their joint effects on mortality and sublethal endpoints for the moth *Mamestra brassicae*. The effects of pesticide exposure on sub-lethal endpoints over the life-cycle was simulated using energy budget TK/TD models from the DEBtox family. The model describes how organisms acquire and use energy for maintenance, growth, and reproduction, and how toxicants impact these processes. The approach could reliably simulate growth and survival of control and exposed moth larvae. The threshold for effects on survival was 0.92 mg/kg for cypermethrin and 2.78 mg/kg for prochloraz, and the threshold for energy budget (growth) was 1.7 10^− 5^ mg/kg for cypermethrin and 0.002 mg/kg for prochloraz. Prediction of mixture effects using additive assumptions frequently underestimated observed effects, indicating a pattern indicative of synergism, especially in lower concentration cypermethrin exposures. The success of the overall experimental and modelling approach, support the further applications of the methods and models used here for pesticide testing for other lepidopteran species. This approach may help improve the ecological risk assessment for pesticide mixtures.

## Introduction

Laboratory toxicity testing is often questioned for simulating exposure scenarios not applicable to the field by focusing on dose-response analysis at only a single time point. Although such analysis is valuable for understanding aspects of the survival effect, traditional models, such as dose-response analysis, often fail to capture the broader, more complex impacts of toxicants over time, particularly on non-lethal life-cycle traits such as reproduction and growth.

Toxicokinetic/toxicodynamic (TK/TD) models, such as the General Unified Threshold Model of Survival (GUTS), have been shown to successfully model the mortality of organisms with an added temporal dimension (Baas et al. 2010). While TK/TD models are valuable for understanding survival dynamics, GUTS cannot simulate the sublethal effects of chemicals, e.g. effects on growth and reproduction. These sub-lethal endpoints are clearly important for populations. For example, longer development times between instars in insects can reduce important population parameters, such as the number of broods per year (Hassold and Backhaus 2009; Wang et al. 2009). This thereby necessitates a modelling approach that captures both acute and chronic effects of toxicants over an organism’s life cycle.

Energy apportionment models, such as those based on Dynamic Energy Budget (DEB) theory are designed to represent the rates at which an organism gains and uses energy for vital processes, such as assimilation, maintenance, growth and reproduction (Kooijman 2010). By integrating energy budget and TK/TD models, it is possible to simulate the effects of chemical exposure on energy resources and life-cycle parameters. Models within the DEBTox family are the most common and well established integrated energy budget TK/TD models used in ecotoxicology (Kooijman 2010; Jusup et al. 2017). Using DEB-TKTD models, it is possible to simulate how the uptake of toxicants disrupts life-cycle processes by affecting the organism energy accrual and use. Further, DEB-TKTD models simulation can be used to make assumptions regarding which physiological process (also known as the physiological mode of action, pMoA), e.g. assimilation, growth, reproduction or maintenance, is most impacted by the toxicant (Jager et al. 2014a; Ashauer and Jager 2018). Such, DEB-TKTD models have been widely used in ecotoxicology, including for modelling mixture effects (Baas et al. 2010; Jager et al. 2014b; Margerit et al. 2016; Bart et al. 2021, 2022; Hansul et al. 2024).

Mixture toxicity effects can be classically attributed as additive (the sum of effects), synergistic (joint action greater than the sum of additivity), or antagonistic (joint action less than the sum of additivity). Previous studies have shown that mixtures that include a pyrethroid and an azole fungicide are commonly synergistic (Cedergreen 2014), with effect sizes for this outcome of up to 50 fold (Kretschmann et al. 2015; Gottardi et al. 2017; Gottardi and Cedergreen 2019). Several studies in bees have identified synergism between pyrethroids and azole fungicides for both mortality and sublethal endpoints (Papaefthimiou and Theophilidis 2001; Wang et al. 2020; Belden 2022). However, to date, there are no studies that detail this synergism in Lepidopteran species by measuring lethal or sublethal toxic effects and the links of these observed effects to underlying TK/TD processes.

Here, we conduct a mixture exposure with the pyrethroid insecticide cypermethrin and the azole fungicide prochloraz to assess their joint effects on mortality and sublethal responses in the cabbage moth *Mamestra brassicae*. Effects on survival, growth, and development were measured from the second instar until pupal emergence. Based on previous observations of synergism for this mixture in other insects, we hypothesised that co-exposure to cypermethrin and prochloraz would lead to a slower development of larvae and increased mortality in mixture treatments surpassing that of the additive effect of the co-exposed individual chemicals. By combining our statistical analysis with DEB-TKTD modelling for impact on growth and survival, we aimed to further study the TK and TD parameters underlying these effects to inform on joint toxicity to support effective pesticide use management.

## Methods

### Bioassay design and pesticide exposures

*M. brassicae* larvae were obtained from a population reared for 40 years on a modified Hoffman’s diet (see Badder et al. 2023). This organism was selected as a species with a distinct larval and adult stage morphology and was used as a model organism to represent lepidopteran species, many of which are pollinators in their adult stage and crop pests as juveniles. Due to the length of time in culture, individuals of this population shared very low genetic variability and as such were expected to have the same response to toxicants and develop at a very similar rate with uniform culture conditions. Further, the culture was synchronised to allow egg collection three times per week. As such all eggs used in this experiment were laid within a 24 h period. Eggs were collected 12 days prior to the experiment and raised at 20 °C ± 2 °C under a 16:8 light: dark regime. This period allows five days for hatching and a further seven days of growth before the larvae were used for testing, as pilot studies had shown poor survival if larvae were handled before 2nd instar. Therefore, 2nd instar larvae selected 7 days post-hatch were used for all exposures.

Individual larvae were exposed to a range of treatments of cypermethrin, prochloraz and mixtures of these two pesticides. These two chemicals were selected due to evidence of synergistic interactions in previous acute tests with *M. brassicae* (unpublished data) and several other sources that report greater than additive effects of cypermethrin and prochloraz tested in combination (Bart et al. 2021, 2022; Cedergreen et al. 2017; Gottardi and Cedergreen 2019). High purity analytical standards (> 99%) both chemicals were purchased from Sigma Aldrich (Poole, UK) for use for all dosing. An initial range finder study was carried out to determine optimal concentrations for toxicity testing. From these results, a concentration range was selected for both single chemicals and the mixture that would cover the expected sublethal response ranges, i.e. the highest dose of cypermethrin would cause 100% mortality and the lowest concentration no mortality (Table 1) .

**Table 1:** Concentrations of Cypermethrin, Prochloraz and the mixture Cypermethrin and Prochloraz in combination used in the growth assay

Cypermethrin (mg/kg)	Prochloraz (mg/kg)	Cypermethrin and Prochloraz (mg/kg)
0.01	1	0.01 + 1
0.05	5	0.05 + 1
0.25	10	0.05 + 5
1.25	20	0.25 + 1
6.25	40	0.25 + 5
		1.25 + 10

To prepare solutions for dosing, the two pure pesticides were solubilised in acetone and diluted with further solvent to give a stock for each test concentration. To provide a spiked exposure medium, the modified Hoffman’s diet was heated and the pesticide stocks added while the diet was still in a liquid state (at ~ 60 °C), to give the required test concentration. A volume of 10 ml of dosed diet was dispensed into an individual round 25 ml plastic pot (53 mm diameter, 27 mm height) to provide the exposure medium for each test replicate. Although each chemical was exposed to temperatures of around 60 °C for a few minutes during medium preparation, heat stability data for both indicates that degradation should be minimal over this very short time scale, although longer incubations would result in hydrolysis of cypermethrin (Lin et al. 2005). To ensure any observed toxicity effects were not related to the presence of residual solvent, the control treatments were always spiked with the same volume of acetone as used in all of the pesticide treatments. In total, for the full experiment, 17 treatments were used, including a control, 5 cypermethrin only treatments, 5 prochloraz only treatments, and 6 mixture treatments.

For each tested condition, 15 individual larvae were exposed singly in plastic pots containing the appropriately spiked diet (*n* = 255). To start the exposure, the 7-day-old individual 2nd instar larvae were weighed and added to the diet surface. All individual larvae were then kept in an incubator in the dark at a constant 20 °C. At the start of every week, fresh diet was prepared and spiked with the required concentration to ensure a more consistent exposure in case of pesticide degradation and also to prevent issues associated with fungal growth on the diet surface. Larvae were transferred to this new diet surface to continue the exposure under *ad-libitum* feeding, meaning that dietary restriction never affected larval growth or development. As a quality control we planned to stop the test if > 20% control mortality occurred, although ultimately this threshold was not reached.

At day 0, 2, 4, 7, 9, 11, 14, 16, 18, 21, 23, 25, 28, 30, 32, 35, 37 and 40, the larvae were taken from the diet surface and checked for survival, weight and development stage. Larvae were recorded dead if there was no movement after five seconds stimulation with a paintbrush. Dead larvae were disposed of immediately and their day of death and instar stage recorded. Survivors were weighed to monitor growth to the 6th instar and, thereafter, their weight loss during preparation for pupation. When reached, day and weight at pupation was recorded and the pupa sexed. Unsuccessful pupations, e.g. attributed to arrested pupal ecdysis, or incomplete shedding of the cuticle, were recorded (Krishnan et al. 2021). After 40 days of exposure, all control larvae had pupated for at least 10 days and the experiment was stopped. Any larvae that had not pupated by day 40 were excluded from analysis, and no data on pupal success was gathered for these individuals. All excluded larvae were in either the 1.25 mg/kg cypermethrin or 1.25 mg/kg + 10 mg/kg cypermethrin and prochloraz treatments. These treatments were, therefore, excluded from the pupation analysis.

## Statistical analyses and DEB-TKTD modelling

The average day of moult to each instar was recorded and tested for significant difference between treatments by ANOVA, with Tukey’s test used to identify significant differences between treatment for each single chemical and the mixture as separate exposures. Average day of moult was similarly compared between treatments. Analysis of the time to pupation data was conducted with GLM and post hoc Tukey’s test. Data on pupation failure was analysed using a Chi-square non-parametric method. Number of larvae reaching pupation varied per treatment group, reflecting that some larvae died during the growth phase, meaning sample sizes per group ranged from seven to thirteen.

DEB-TKTD analysis used the simplified DEBkiss model. This formulation was developed and is fully presented in Jager and Zimmer (2012) and Jager (2020). In the model, food is taken up by the caterpillars and a fraction (κ) of the assimilated energy is used for the soma (growth and somatic maintenance), whereas the remainder (1 − κ) is used for maturation (in juveniles) and maturity maintenance, and reproduction (in adults). Here, only the growth phase (juveniles) of the life cycle was considered, and all model simulations ended once the larvae entered pupation. Therefore, only the DEB branch where assimilated energy is used for growth and somatic maintenance was considered.

In contrast to other simplified DEB approaches, DEBkiss builds upon an explicit mass balance, and excludes the distinction of biomass in a structure and reserve compartment: all biomass is treated as “structure”, an acceptable simplification for invertebrates (Jager et al. 2013). The assimilation energy flux is written as:

$$\:{J}_{A}\left(t\right)=f\:{J}_{Am}^{a}{\:L}^{2}\left(t\right)$$

Where *J*_*A*_ [ma d^− 1^] is the mass flux for assimilation, $\:{J}_{Am}^{a}$ [ma/(L^2^d^− 1^)] is the area-specific assimilation rate at maximum food, f [-] is the scaled functional response which reflects food availability (between 0, no food, and 1, *ad libitum* feeding condition), L^2^ [mm^2^] is the volumetric length. The maintenance energy flux is described as:

$$\:{J}_{M}\left(t\right)=\:{J}_{M}^{v}{\:L}^{3}\left(t\right)$$

Where *J*_*M*_ is the mass flux for maintenance, $\:{J}_{M}^{v}$ [ma/(L^3^d^− 1^)] is the volume-specific costs for maintenance, and L^3^ [mm^3^] is the volumetric length. The mass flux for structure is described as:

$$\:{J}_{V}\left(t\right)={y}_{VA}\left(k{J}_{A}\right(t)-{J}_{M}(t\left)\right)$$

Where *J*_*V*_ is the mass flux for structure (i.e., =growth), y_VA_ is the yield of structure on assimilates (growth), k (-) is kappa, the part of the energy used for growth and somatic maintenance. The remaining energy (1-kappa) is used for maturation in juvenile stage and egg production in the adult stage, although here we did not include this aspect, as only the larvae growth period was considered.

Traditional DEB models require the parameter of length to determine growth. However, this measurement was difficult to obtain with cabbage moth larvae due to their tendency to curl when disturbed. Therefore, weight was used as a more reliable measurement endpoint in this species. *M. brassicae* larvae are isomorphic, meaning that their body shape does not change during growth. The implication of this is that volume is proportional to cubed length, therefore, the cubic root of the body weight can be used to calibrate the model (Bart et al. 2019).

As presented in Badder et al. (2023), M. *brassicae* present an exponential growth curve when measured either in biomass or structural length. This phenomenon, described in other insect species, has been explained by type M metabolic acceleration as presented in Kooijman (2014). This results in the progressive increase of the maximum food assimilation rate $\:{J}_{Am}^{a}\:$and energy conductance during this period due to their multiplication by an acceleration factor *S*_*M*_. This factor evolves from *S*_*M*_ = 1 at birth to reach $\:{S}_{M}=\frac{{L}_{p}}{{L}_{b}}$ at pupation. Between these two stages, it evolves along with length: $\:{S}_{M}=\frac{L}{{L}_{b}}\:$and the Assimilation flux is now described as:

$$\:{J}_{A}\left(t\right)=f\:{J}_{Am}^{a}{{S}_{M}\:L}^{2}\left(t\right)$$

Finally, the start of pupation depends on hormonal processes and so is not related to energy fluxes. We, therefore, assumed that pupation starts once individuals reach a certain size, *L*_*p*_, and the growth stops; if *L* > *L*_*p*_, *J*_*V*_ = 0. A mortality background *h*_*b*_ (d^− 1^) is also added to account for mortality not related to the toxic compound. On these bases, altogether the parameters, $\:{J}_{Am}^{a}$, *f*, $\:{J}_{M}^{v}$, *y*_*VA*_, *S*_*M*_, *k*, *L*_*p*_, *h*_*b*_, describe the physiological processes in the DEBkiss-TKTD model.

The TK-TD component of the overall model accounts for the accrual of, and recovery from, damage (toxicodynamics, TD), which forms due to the bioaccumulation, distribution, biotransformation, and elimination of the chemicals in the organism (toxicokinetics, TK). From the study there was no information on body residues (measurements or predictions) available. Therefore, the TK and TD part are combined into a one compartment model linking the external concentration to the damage, over time), which takes the form:

$$\:\frac{d{D}_{f}}{dt}={k}_{d}({C}_{f}-{D}_{f})\:\:\:\:\:$$

where *D*_*f*_ (mg/kg) is the damage level (scaled by the external concentration in the food), *C*_*f*_ (mg/kg) is the total concentration in the food and *k*_*d*_ (d^− 1^) is the dominant rate constant describing the dynamics of the “scaled” damage and represents the one-compartment approximation of the “true” two-compartment behaviour (TK and damage dynamics). From the scaled damage level, a dimensionless stress level is calculated (Eq. 1):

1 $$\:s={b}_{b}\text{max}\left(0,{D}_{s}-{z}_{b}\right)$$

Where *s* is the stress level, *b*_*b*_ (mg/kg) is the effect strength on the energy budget, and *z*_*b*_ (mg/kg) is the damage threshold for effects on the energy budget. The stress modifies the value of one or more DEBkiss model parameters (Jager 2020). Only the growth phase of the life cycle was considered; therefore, three metabolic processes can be affected by the chemical: assimilation $\:{J}_{Am}^{a}\left(1-s\right),$ maintenance $\:{J}_{M}^{v}\left(1+s\right)\:$and growth $\:{y}_{VA}/(1+s)$), or a combination of these. The affected metabolic process is generally referred to as a physiological mode of action (pMoA); A, M, G.

Finally, the model assumes the same damage type affects both sublethal and lethal toxicity. To model survival the stochastic death module from the GUTS framework is added to the model :

2 $$\:h={b}_{s}\text{max}\left(0,{D}_{s}-{z}_{s}\right)$$

3 $$\:\frac{dS}{dt}=-\left(h+{h}_{b}\right)\:S\:with\:S\left(0\right)=1$$

Where *S* is the survival probability over time, *h* (d^− 1^) is the hazard rate, *b*_*s*_ (mg/kg d^− 1^) is the effect strength on survival (also known as killing rate, *b*_*w*_, in the GUTS framework), and *z*_*s*_ (mg/kg) is the damage threshold for survival. Together, *k*_*d*_, *b*_*b*_, *z*_*b*_, *b*_*s*_ and *z*_*s*_ form the five toxicological parameters of the DEBkiss-TKTD model. For the growth, a stress level is calculated for each chemical, and it modifies the value of one or more DEBkiss model parameters. If the two chemicals share the same pMoA, the stress levels are multiplied (e.g., for pMoA growth $\:{y}_{VA}/\left(\left(1+sA\right)*\right(1+\:sA)$)).

The model can be calibrated with different feedbacks on damage dynamics that include surface: volume on uptake, surface: volume on elimination, growth dilution and losses with reproduction. As we used only the larval stages of *M. brassicae*, reproduction was not included as a feedback option, but combinations with the other three feedbacks were tested and the best fit, assessed by MLL and R2 value, was selected for the cypermethrin and prochloraz single chemical exposures.

Once the DEB-TKTD model is calibrated on single exposures, the mixture effects can be predicted assuming additivity. The prediction of the mixture interaction was performed using the GUTS_MIX Matlab package (Bart et al. 2021). Briefly, this model is first calibrated for effects using the single chemical data, then using the parameters obtained from these calibrations, a prediction of the mixture effect over time is made and plotted against the observed data. If observed effects exceed those from the additive model, then this can be interpreted as an underestimation of actual effect indicative of synergy; effects less than the additive model indicate an overestimation of actual effects, indicative of antagonism; an accurate simulation indicates additivity.

All model calculations were performed in Matlab 2021 with the BYOM v.6.0 modelling platform (http://www.debtox.info/byom.html), with the package DEBtox2019, modified to account for the metabolic acceleration of *M. brassicae*. The physiological parameters of the model were fitted first to the control condition. Next, the toxicological parameters were fitted to the entire dataset (i.e., on survival, growth and reproduction data together), keeping the physiological parameters fixed to their best value. Finally, with calibrated models (the best parameter set associated with the best pMoA, and feedbacks on TKTD if relevant), the mixture effect was predicted and visually assessed to see if synergism or antagonism could be identified against the additive model predictions.

## Results

### Mortality and growth in the single chemical exposures

**Fig. 1:**
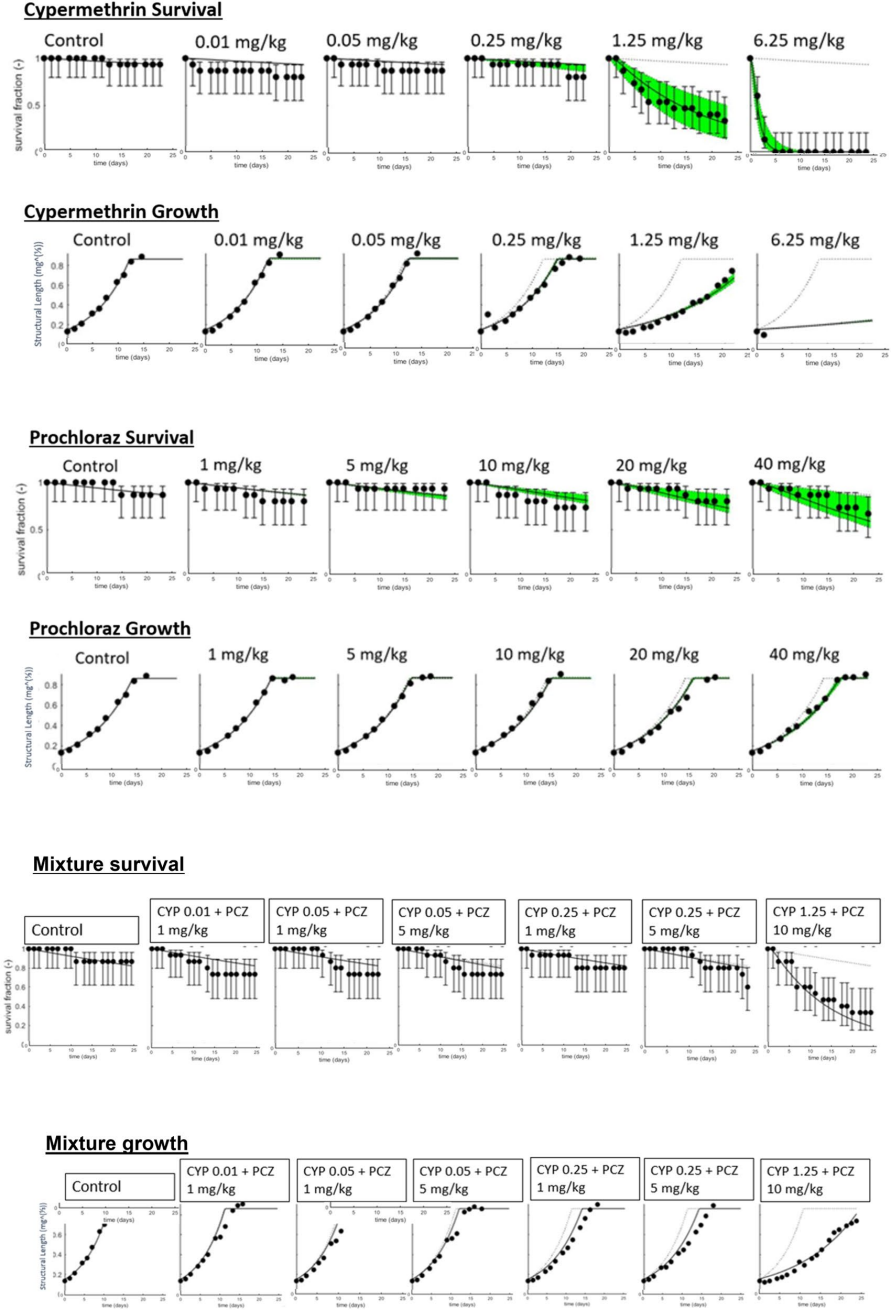
Survival and growth of larvae exposed to cypermethrin (top two graphs), prochloraz (middle two graphs) and their mixture (bottom two graphs); for survival (first row of graphs in each pair), the dotted line indicates background mortality, bold lines the model fits, and points the actual survival data (with error bars showing Wilson score confidence); for growth (second row of graphs in each pair), points indicated measured data and solid lines the model fits, the dotted line indicates control growth with green areas the 95% confidence intervals of model fits

Control mortality in the cypermethrin only experiment was 13.3% over the 40 d duration of exposure, below the upper limit of 20% mortality that we used as a validation threshold for the test. At 6.25 mg/kg, the highest concentration of cypermethrin tested, all larvae were dead after 7 d. A proportion of larvae survived for the full 40 d at all other tested concentrations. The treatments of cypermethrin selected, thus, represent a range of exposure levels covering full, partial and no additional mortality above background (Fig. 1 top).

The DEB-TKTD model accurately simulated larval growth. Fits indicated that the best pMoA for cypermethrin growth effects was an increase in growth energy cost (Table 2) but any feedbacks did not improve the fit so were not used. All control larvae stopped gaining weight in preparation for pupation by day 23 (Fig. 1 top). Arrest of growth was also found by this time for larvae exposed to 0.01 and 0.05 mg/kg cypermethrin. Growth curves for the 0.25 mg/kg and 1.25 mg/kg cypermethrin treatments were shallower than for controls. All larvae exposed to 1.25 mg/kg cypermethrin continued growth after 40 d, a time by which all other treatments had pupated. In this treatment, growth was only modelled up to 25 d to provide consistency with the lower concentration treatments. At 6.25 mg/kg all larvae were dead at 7 d. The weight change data modelled over this initial period indicated only a small amount of growth in this treatment.

**Table 2:** Calibrations of pMoA’s for *Mamestra brassicae* larvae exposed to treatments of Cypermethrin and prochloraz, the blue bar represents the best fit, due to lowest MLL and AIC values

Chemical	pMoA	MLL	AIC	R2 Surv	R2 Body Length
Cypermethrin	Growth	203	416	0.96	0.982
Cypermethrin	Growth + Maintenance	203	416	0.96	0.982
Cypermethrin	Assimilation + Growth	213	435	0.96	0.978
Cypermethrin	Assimilation + Maintenance + Growth	213	435	0.96	0.978
Cypermethrin	Assimilation + Maintenance	219	447	0.96	0.973
Cypermethrin	Assimilation	218	447	0.961	0.974
Cypermethrin	Maintenance	286	582	0.96	0.616
Prochloraz	Growth + Maintenance	189	389	0.572	0.991
Prochloraz	Growth	190	389	0.534	0.991
Prochloraz	Assimilation + Growth	190	391	0.612	0.991
Prochloraz	Assimilation + Maintenance + Growth	191	391	0.624	0.991
Prochloraz	Assimilation + Maintenance	191	393	0.623	0.99
Prochloraz	Assimilation	192	394	0.539	0.99
Prochloraz	Maintenance	230	472	0.603	0.968

G is growth, M is maintenance and A is assimilation, the lighter blue bars shows equal or similar MLL or AIC values

For prochloraz, effects on larval survival were found (Fig. 1 middle), with effects at 40 mg/kg apparent from 20 d. No significant effect was found on mortality at 10 and 20 mg/kg. Exposure at these levels, thus, provided an opportunity to assess how exposure to prochloraz affects growth and development traits when there is both partial and no mortality.

The DEB-TKTD model was able to simulate growth in the control and prochloraz exposed larval over 25 d, the main growth period. The best pMoA combination for prochloraz effects on larvae growth was an increase in energy cost associated with an increase in maintenance cost (MLL = 189), although this fit is very similar to effects on growth only (MLL = 190) indicating the dominant pMoA relates to growth (Table 2). Again, feedbacks did not improve the fit, so were not used. All but one larva exposed to prochloraz reached the 6th instar by day 22, but deviations in growth from the controls was observed. These prochloraz effects were not as pronounced as for cypermethrin (compare Fig. 1 middle to Fig. 1 top). Nonetheless, at exposure concentrations of 10 mg/kg and above, there was an increasing deviation of fitted growth from the model for the control (shown as a visible dotted line in Fig. 1 middle, second row) with increasing prochloraz concentration.

DEB-TKTD models were fitted to the observed survival in time, growth and pupation time data to provide a pMoA and associated set of TK/TD and energy budget related physiological parameters for each chemical and the mixture. Both cypermethrin and prochloraz tested concentrations exceeded the estimated threshold for effects on energy budget (*zb*), predicting an effect on growth proportional to the effect strength energy budget (*bb*) and the concentration exceeding the *zb* value (Table 3). In both cases the estimated *zb* value approached the lower model boundary, which reflects that growth effects were evident even at the lowest tested concentrations. This outcome indicates that the true threshold for effects is likely very close to zero, rather than representing a failure of the model. On the contrary, the model accurately captured the observation that no clear no-effect level was present in the dataset, strengthening confidence in its predictions. The threshold for effects (*z*_*s*_) on survival was lower for cypermethrin than prochloraz (0.92 vs. 2.78 mg/kg respectively). The effect strength for survival (*b*_*s*_) was also higher for cypermethrin than prochloraz (Table 3), consistent with a higher concentration dependent effect on survival for the insecticide (compare survival effects with treatment in Fig. 1 top with Fig. 1 middle).

**Table 3:** Parameter values, with 95% confidence intervals, of the DEB-TKTD models

Symbol	Description	Value	Unit
*DEBkiss parameters*			
$\:{J}_{Am}^{a}$	Maximum area-specific assimilation rate	0.0056 (0.005–0.0117)	mg/mm^2^/d^− 1^
$\:{J}_{M}^{v}$	Volume-specific maintenance costs	0.0101 (0.0037–0.0996)	mg/mm^3^/d^− 1^
*L*_*0*_	Initial structural length	0.036	mg ^(1/3)^
*y*_*VA*_	Yield of structure of assimilates (growth)	0.8 (fixed)	-
$\:f$	Scaled functional response (0–1)	1 (fixed)	-
*S*_*M*_	Metabolic accelaration factor	-	L(t)/L_0_
*k*	Kappa	0.9 (fixed)	-
*L*_*p*_	Structural length at which growth is ceased, start moulting	0.86	mg ^(1/3)^
*h*_*b*_	Mortality background	0.0021 (0.0018–0.0027)	d^− 1^
*Toxicological parameters for cypermethrin*			
*k*_*d*_	Dominant rate constant	1.32 (0.67–10)	1/d
*Z*_*b*_	Threshold energy budget	1.7 × 10^− 5^ (1.7 × 10^− 5^ − 0.008)	mg/kg
*b*_*b*_	Effect strength energy-budget	0.95 (0.86–1.03)	kg/mg
*Z*_*s*_	Threshold survival	0.92 (0.2–1.08)	mg/kg
*b*_*s*_	Effect strength survival	0.11 (0.05–0.19)	kg/mg/d
*Toxicological parameters for prochloraz*			
*k*_*d*_	Dominant rate constant	0.21 (0.03 -10)	1/d
*Z*_*b*_	Threshold energy budget	0.002 (0.002–2.846)	mg/kg
*b*_*b*_	Effect strength energy-budget	0.007 (0.005–0.027)	kg/mg
*Z*_*s*_	Threshold survival	2.78 (0.002–37.77)	mg/kg
*b*_*s*_	Effect strength survival	0.0004 (9.2 × ^10−5^ − 0.01)	kg/mg/d

Physiological mode of action for Cypermethrin and prochloraz: increase of the growth energy cost

### Mortality and growth in the mixture exposures

The DEB-TKTD models for the single chemicals were used to predict the joint effects of cypermethrin and prochloraz on larval survival, assuming no interaction. Compared to the mixture effect model predictions, the observed data indicated no consistent evidence of synergism in the 1.25 cypermethrin + 10 mg/kg prochloraz treatment, where the observed data closely accorded with the model predictions, indicating additivity (Fig. 1 bottom). The collected data suggest that synergism was indicated by a model underprediction of the observed mixture effect on survival seen for 0.25 mg/kg of cypermethrin when exposed to both test concentrations of prochloraz (1 mg/kg and 5 mg/kg). However, all data points are within data uncertainty suggesting no statistically significant synergistic impact on larval survival.

**Fig. 2:**
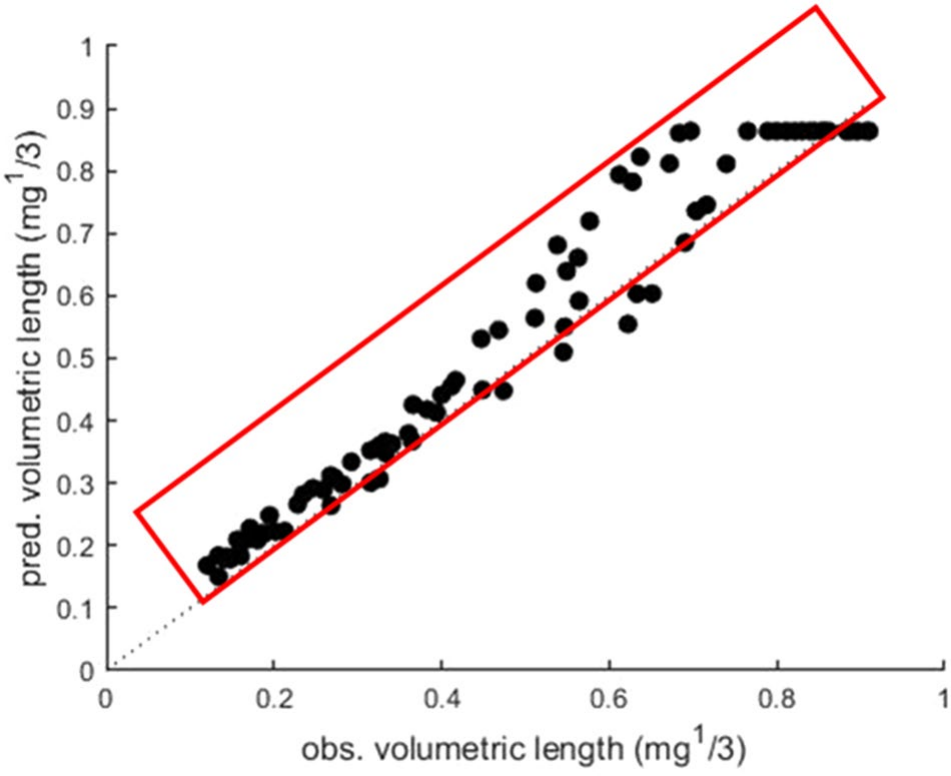
Predicted vs. Observed effects of cypermethrin and prochloraz in mixture on *M. brassicae* growth; the red rectangle denotes the under estimation of the effects on growth predicted by the model compared to the true observed effects

To better assess the potential synergism between the two chemicals, we plotted the correlation between the observed versus predicted weights to allow us to identify the pattern of distribution of measured versus modelled growth across all individuals (Fig. 2). Modelled additive effects on larval growth consistently lie above the observed volumetric length values (Fig. 2), indicating that, due to synergism, actual body sizes do not generally reach those predicted based on additivity.

Cypermethrin had a significant concentration related effect on development time as indicated by the day of moult values (ANOVA, *p* < 0.001). Larvae exposed to 0.25 and 1.25 mg/kg of cypermethrin developed to each instar at significantly later timepoints than controls (Tukey’s test *p* < 0.001)(Table 4). This delay in maturation is particularly prominent for the 4th, 5th and 6th instars (Table 4 top).

**Table 4:** Average day of moult to each instar (i) in *Mamestra brassicae* larvae exposed to Cypermethrin (top), Prochloraz (middle) and their mixture (bottom); columns in tables indicate data for different instars (i3, i4, i5, i6); letters within each table indicate significant differences between concentrations for each instar (*p* < 0.05)

Cypermethrin (mg/kg)	Average Day of Moult to Each Instar			
	i3	i4	i5	i6
Control	2.8 A	5.5 A	10.6 AB	15.5 A
0.01	3.5 A	6.3 AB	10.8 AB	15 A
0.05	3.1 A	6.1 AB	10.3 A	15.8 A
0.25	3.7 AB	7.1 B	11.8 B	25 B
1.25	5.67 B	10.8 C	19.8 C	31.25 C
Prochloraz (mg/kg)				
Control	2.8 A	5.5 A	10.6 AB	15.5 A
1	3.5 A	6.2 AB	9.7 A	15.7 A
5	2.9 A	5.9 AB	10.7 AB	15.5 A
10	2.7 A	5.5 A	12.3 B	17.5 AB
20	3.7 A	7.6 B	14.8 C	19.8 BC
40	3.2 A	5.6 A	15.6 C	22 C
Cypermethrin +Prochloraz (mg/kg)				
Control	2.8 A	5.5 A	10.6 A	15.5 A
0.01 + 1	3.3 AB	6.2 AB	12.8 AB	20 B
0.05 + 1	3.7 AB	12.3 DE	16.1 C	20.8 BC
0.05 + 5	3.6 AB	10.5 CD	15.2 BC	20.9 BC
0.25 + 1	4.3 B	10.6 CD	17.2 C	23.5 BC
0.25 + 5	3.5 AB	8.8 BC	16.3 C	23.8 C
1.25 + 10	7.4 C	13.6 E	22.4 D	38.3 D

A significant difference in the time between moults was also found for prochloraz exposed larvae (ANOVA, *p* < 0.001). Slower development was seen at concentrations of 20 mg/kg and higher. These effects of prochloraz exposure were mainly seen in the later instars. Thus, moult times for 5th and 6th instars were significantly later (Tukey’s test *p* < 0.001) than for controls at 20 and 40 mg/kg, the two highest prochloraz concentrations, but not for instars 3 and 4, e.g. in the 40 mg/kg treatment (Tukey’s test, *p* > 0.05, Table 4 middle).

Mixture treatments showed significant difference in moult times for all instars (3rd instar *p* < 0.001, 4th instar *p* < 0.001, 5th instar *p* < 0.001, 6th instar *p* < 0.001). By the last instar all treatments differed significantly (Tukey’s test, *p* < 0.001) from controls (Table 4 bottom). For the two 0.05 mg/kg cypermethrin mixture treatments, significant changes in moulting time occurred as early as instar 4 and continued in later instars. The extent of this effect again differs from the single cypermethrin 0.05 mg/kg treatment, for which no significant effects on moult time were seen (Table 4). With the exception of instar 3 for the 0.25 CYP + 5 PCZ mg/kg treatments, all 0.25 mg/kg CYP with PCZ had a significant effect (Tukey’s test, *p* < 0.001) on development in all instars. This is consistent with the slower larval development seen in these treatments.

Pupation success was recorded alongside any incidences of arrested pupal ecdysis. In the highest tested cypermethrin concentration, no larvae reached a size appropriate for pupation within 40 days. Hence, it is uncertain whether there could be effects on pupation at this concentration after further growth. No significant difference in incidences of pupation failure were found for the different concentrations of cypermethrin (*X*^*2*^(3) = 1.4 *p* > 0.05), prochloraz (*X*^*2*^(5) = 5.52 *p* > 0.05) or the mixture respectively (*X*^*2*^(15) = 22.1 *p* > 0.05).

**Fig. 3:**
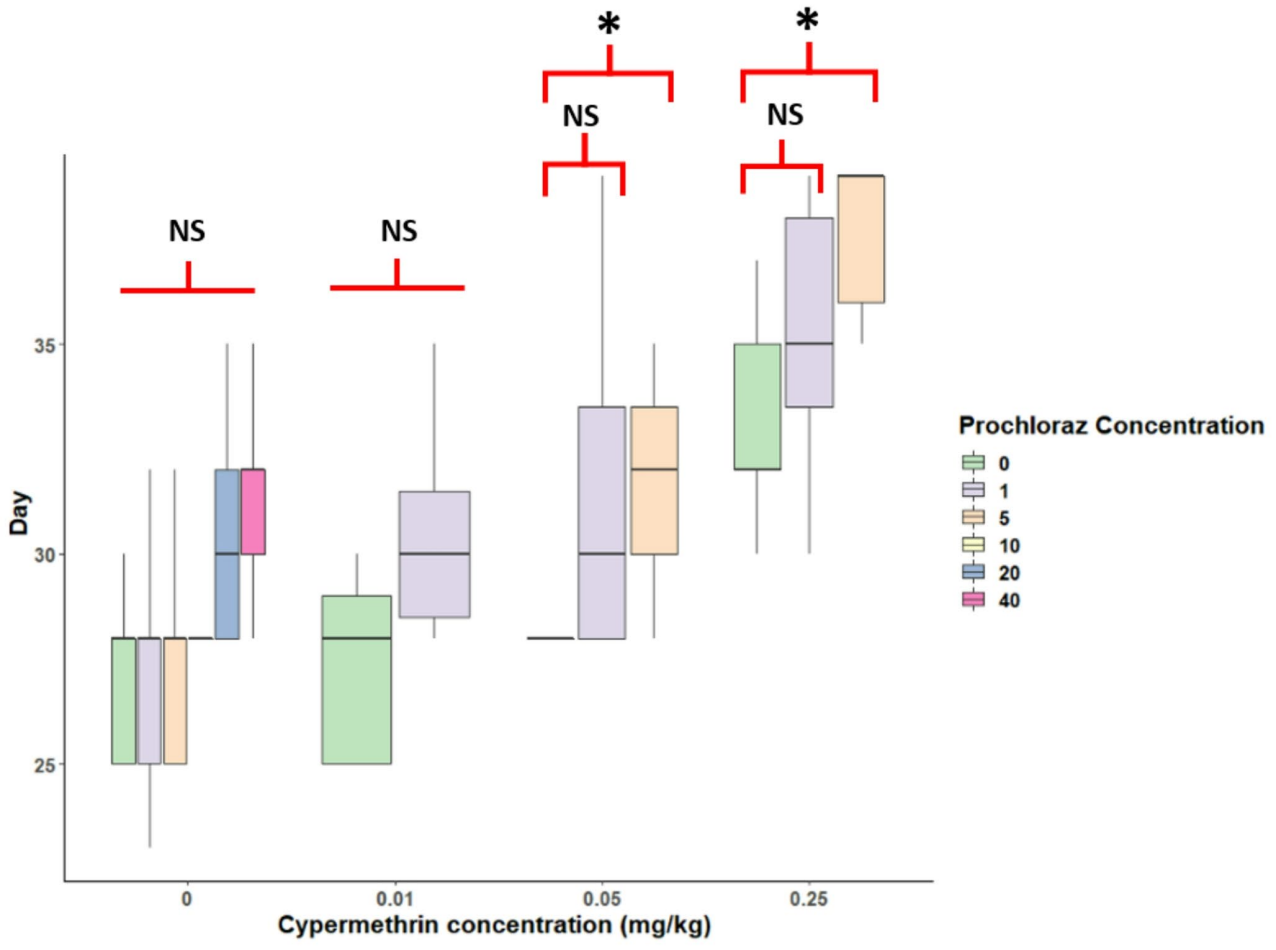
Mean (bold line) 75th percentile (upper and lower box limits) and 95% confidence intervals (vertical lines) of days to pupation for larvae exposed to cypermethrin, prochloraz and their mixture. Significant effect indicated by GLM are showed, *p* < 0.05, NS = not significant

A statistically significant difference in day to pupation was found between both single chemical and mixture treatments (ANOVA, cypermethrin *p* < 0.001, prochloraz *p* = 0.001, mixture *p* < 0.05). Cypermethrin significantly increased pupation time in the 0.05 and 0.25 mg/kg exposures compared to controls (ANOVA, *p* < 0.001). Further, all treatment groups that contained prochloraz took longer to pupate than those with cypermethrin alone (Fig. 3). This increase appeared concentration dependent. Thus, significant effects on time to pupation (Tukey’s test, *p* < 0.05) were only identified in mixtures at 0.05 and 0.25 mg/kg cypermethrin in the presence of 5 mg/kg, but not 1 mg/kg, of prochloraz (Fig. 3). The last larvae to pupate within the 40 day experimental period were those in the 0.05 CYP + 5 mg/kg PCZ mixture. As prochloraz and cypermethrin both significantly affected development and pupation times, this effect may reflect the additive effect of these two chemicals, rather than necessarily indicating synergism.

## Discussion

The assessment of chemical toxicity in laboratory settings has long faced criticism for its limited scope and lack of environmental realism, often relying on short-term exposure studies that fail to capture the dynamic nature of organism responses. This lack of a temporal aspect in standard testing highlights the importance of dynamic based approaches, such as those in the DEB-TKTD family of integrated energy budget and TK/TD models, to more accurately reflect the temporal aspects of toxicity. Here we use DEB-TKTD based thinking and tools to investigate how exposure to cypermethrin and prochloraz and their mixture impacts not only on lethal outcomes, but also on sublethal endpoints such as growth and development in the moth *M. brassicae*.

In the absence of chemical stressors, larvae use assimilated energy for metabolism, growth or maturation. On exposure to a toxic stressor, there is the potential that a greater proportion of overall energy would be required to maintain metabolism through damage repair, or that the toxicant leads to greater energy cost for growth or maturation/reproduction. It has been reported that this increase in costs to maintain somatic tissues also in turn reduces energy available for growth (Soetaert et al. 2007). In this study, larvae exposed to cypermethrin concentrations of 0.25 mg/kg and above clearly exhibited delayed growth and extended development times from the 4th instar onward. At the highest exposure level of 1.25 mg/kg, time to moult was increased by approximately 50% compared to controls for all instars.

The DEB-TKTD modelling analysis indicated that the threshold energy budget (*z*_*b*_) for cypermethrin was 1.7 × 10^− 5^ mg/kg. This suggests that even low concentrations of cypermethrin could potentially disrupt growth rate and delay pupation, although it should be noted there is relatively high uncertainty linked to this value. This very low threshold for sublethal effects indicates that cypermethrin might disturb lepidopteran larval energy budgets at realistic off-field concentrations in agroecosystems, leading to resulting delays in pupation that may ultimately affect population dynamics. This highlights the potential physiological effects of this insecticide for target and also non-target species. The dominant rate constant (*k*_*d*_*)* represents the combined effect of metabolism and excretion to remove the chemical from the body. The value of this parameter was intermediate in cypermethrin exposure (1.32 d^− 1,^), which means toxicokinetic-toxicodynamic processes were neither slow nor fast (Jager and Ashauer 2018).

To fully understand the pMoA of cypermethrin, knowledge of the primary biochemical mechanism of action may help to understand the linked energy budget impacts. Cypermethrin exerts its toxic effects on insects by binding to the voltage-gated sodium channel, converting it from its active ion-conducting state to an inactive and non-conducting condition (Davies et al. 2007). As a result, nerve cells become depolarised leading to paralysis (Field et al. 2017). For cypermethrin, the best fitting pMoA was an increase in energy costs for growth. However, it is important to highlight that differences between the model fit of cypermethrin (presented in Table 2) for growth alone and each of growth-maintenance, growth- assimilation and growth-assimilation-maintenance were marginal. This comparability indicates that while growth appears to be the main pMoA of cypermethrin, disturbance of assimilation or maintenance pathways may also contribute to toxicity. In the simplified DEB model, a pMoA of growth implies that the cost of production of somatic tissue increases with exposure. In other words, exposed larvae have a greater expenditure for the production of new biomass than controls (Jager et al. 2010). This is in contrast to a pMoA of maintenance which implies that the somatic maintenance cost is increased and one of assimilation which implies there is a decrease in the efficiency of energy uptake (Hansul et al. 2024). Ultimately, it may be the case that although growth may be a primary pMoA, the primary DEB parameters, although nominally independent of each other, are in reality connected by an underlying physiological process that inherently includes linked biochemical pathways. In this case, a more detailed data-set tested with a greater number of treatment levels would be required to reliably identify the specific pMoA.

Chemicals with the pMoA on growth energy cost have been reported to impact the ability to synthesise amino acids and proteins, due to inhibition of ribosomal activity, the site of protein production. A study by Swain et al. (2010) identified disruption to the KEGG protein transport pathway for a chemical (fluoranthene) that also affects costs for growth, suggesting a change in protein metabolism may be responsible for effects through this pathway. From our study, it is not possible to identify how the known mechanism of action of cypermethrin on nerve cells through voltage gated sodium channel interactions impacts the processes that underpin the creation of new somatic tissues, such as protein synthesis. However, it is known that the nervous system plays a critical role in the physiology of metabolism during tissue production. In cypermethrin exposed individuals, sublethal effects of nerve function seemingly impact the ability of larvae to produce new cell structure, potentially either by changing production rates or decreasing the viability of new cells (Jin et al. 2011; Huang et al. 2016).

Azole fungicides, such as prochloraz, are known to disturb processes such as hormone synthesis and neonatal development in both vertebrates and invertebrates through the inhibition of CYP450 mediated pathways (Zarn et al. 2003). It has also been reported that, azole fungicides can delay time between moults of invertebrate species, even completely preventing this process (Hassold and Backhaus 2009). Here, the pMoA for prochloraz could not be confidently determined, but it is likely that growth energy cost is most affected. The nature of the observed effects mean it is not feasible to reliably attribute a single cause of death for the prochloraz exposed larvae. However, it was noted that several of the larvae died during or just after moults in which the cuticle was not shed correctly. This phenomenon has been previously reported in crustacean species as a result of prochloraz inhibition of ecdysteroid synthesis during moulting (Hassold and Backhaus 2009). The potential effects on moulting observed here may indicate that prochloraz exposure also inhibits this pathway in lepidopterans. In lepidopterans the prothoracicotropic hormone (PTTH) stimulates moulting (Perez et al. 2007). While there is no evidence of prochloraz mediated inhibition of this hormone leading to effects on somatic growth and development, this could be a hypothesis to investigate in future studies.

Larvae exposed to mixture treatments showed higher mortality and slower growth and development than those exposed to cypermethrin alone without prochloraz. Using the DEB-TKTD it was possible to visualise this effects, which was shown to be consistent across the full growth period. For growth and development, the observed response exceeded that predicted from the additive effects of the two chemicals, suggesting a potential synergistic interaction, although formal statistical testing is needed to confirm its significance. At present, there are no tools in the BYOM modelling platform to propagate the uncertainty of a DEB-TKTD model applied to mixtures, nor is there a tool to assess the significance of synergistic effects. Further work on how to propagate uncertainty in mechanistic models accounting for mixtures is, therefore, required to improve the basis for assessing the strength of evidence for synergism.

The evidence for synergism was clearest in mixtures with lower cypermethrin concentrations, e.g. no evidence of synergism at the highest mixture treatment of 1.25 CYP + 10 PCZ mg/kg. Similar results have previously been reported in a study that used TK/TD models to analyse the joint impact of α-cypermethrin and prochloraz, in which synergism was seen at low but not at higher cypermethrin levels (Cedergreen et al. 2017). The observation of differences in synergism pattern at different exposure levels is consistent with the concept of dose level dependent interaction formalised by Jonker et al. (2005).

Using a lepidopteran as a test species allowed for the exploration of non-standard endpoints, such as pupation rates, but limited the life-cycle study to a single life stage. Owing to their morphologically different life stages and feeding strategies, it was not possible to perform an analysis of adults and juvenile cabbage moths using the same test method. However, lepidopterans such as *M. brassicae* provide a complex physiology for further study in terms of energy flow in the larval and pupal development stages. In the larval stage, energy is taken in as food and used to fuel processes such as maturation and growth. Here, a supply of energy must be built up rapidly in the fat body in preparation for pupation (Merkey et al. 2011; Jiang et al. 2019). Then, as pupa, energy flow is one directional as energy (food) is no longer taken in, but there is a significant metamorphic transformation occurring within the pupa, which requires an energy source (Nestel et al. 2003). Finally, larvae are transformed from caterpillars that primarily prioritise feeding, into flying adults capable of reproduction. In this stage, growth and maturation are not prioritised, in favour of mating and egg laying. For some holometabolic species, energy at this stage is also one directional, as adults do not take in energy as food, but expend energy in gamete and offspring production.

The change in resource allocation in the period prior to pupation could explain the delayed growth and development of the cypermethrin and mixture treatments compared to the controls. In terms of energy, this could be because larvae expend an additional amount throughout their development for detoxification and damage repair. Any such increase would mean that they lack the resources needed for growth and pupation. For example, glycogen is needed for chitin synthesis for cuticle development (Arrese and Soulages 2010; Qu et al. 2021) and in some insect species a spike in glycogen levels has been reported immediately before pupation (Nestel et al. 2003; Sak et al. 2009). Therefore, if glycogen stores are depleted through reduced energy availability as a result of increased growth costs, then formation of new cuticle would be slowed or even prevented during moulting such that entry into pupation may be inhibited. Further work to explore the relationships between pesticide exposure, larval energy status, and pupa ecdysis are recommended, for example, measuring effects over a greater range of concentrations and/or directly measuring energy stores.

Detoxification enzymes such as *CYP450*s and esterases have been reported to be key to the metabolism of cypermethrin. Esterases hydrolyse cypermethrin and *CYP450s* render them into an excretable form that can be conjugated by phase II enzymes (Nakamura et al. 2007; Baek et al. 2010; Muthusamy and Shivakumar 2015). As the MoA of prochloraz is based on inhibition of *CYP450s* involved in sterol synthesis (Henry and Sisler 1984), it is likely that the inhibition of further xenobiotic metabolising endogenous *CYP450s* plays a role in the synergistic interaction between cypermethrin and prochloraz. Indeed, azole fungicides are commonly reported to increase the toxicity of pyrethroid insecticides by inhibiting *CYP450* isoforms essential for the metabolism of lipophilic toxicants such as cypermethrin (Kretschmann et al. 2015; Gottardi et al. 2017; Bart et al. 2021). Of several azoles tested, prochloraz is thought to have one of the greatest potentials for *CYP450* inhibition (Kretschmann et al. 2015; Hassold and Backhaus 2009). This inhibition potency could explain the indicative observations of synergism in the low cypermethrin concentration treatments.

## Conclusion

A criticism of laboratory toxicity testing is the short-term nature of the exposure methods and analysis of a limited range of sublethal end points only at a single time-point (Jager 2011). In order to assess the effects of chemical exposure on an organism, integrated energy apportionment and TK/TD models such as those in the DEBTox family can be used to determine which sub-lethal processes will be impacted most by chemical exposure and the underlying physiological causes. Such data can be of value to provide mechanistic insights and to improve hazard assessment. Here we demonstrate that a simplified version of the DEB-TK/TD model can be applied to simulate chemical effects on a range of larval growth and development traits in the lepidopteran *M. brassicae*. The model successfully simulated growth and survival of larvae exposed to cypermethrin and prochloraz. Predictions of mixture effects using additive assumptions frequently underestimated the observed growth effects of exposure to mixtures of the two pesticides, especially in lower concentration exposures, although these results can only be considered as indicative within the data analysis framework used. These results suggest that cypermethrin and prochloraz mixtures could be frequently synergistic for both lethal and sublethal endpoints in Lepidoptera, a result consistent with observations for other species. The potential of prochloraz to inhibit CYP450s involved in the metabolism of cypermethrin was suggested as the possible cause of this synergism. The success of the overall experimental and modelling approach, supports the further applications of the methods and models used here for pesticide testing for other lepidopteran species.

## Data Availability

Data available from author on request.
